# Maintaining over time Clinical Performance targets on Anaemia correction in unselected population on chronic dialysis at 20 Italian Centres. Data from a retrospective study for a Clinical Audit

**DOI:** 10.1186/1471-2369-10-33

**Published:** 2009-10-24

**Authors:** Silvia Soffritti, Giorgia Russo, Stefano Cantelli, Giuseppe Gilli, Luigi Catizone

**Affiliations:** 1 Division of Nephrology, S. Anna University Hospital, Corso Giovecca 203, 44100 Ferrara, Italy; 2 Office of Medical Statistics, Health Physics Service, S. Anna University Hospital, Corso Giovecca 203,44100 Ferrara, Italy

## Abstract

**Background:**

The Italian and European Best Practice Guidelines (EBPG) recommend a target haemoglobin value greater than 11 g/dl in most patients with Chronic Kidney Diseases. However, it is still difficult to maintain these values at a steady rate. Thus, the main aim of the study was to evaluate, throughout 2005, how many patients steadily maintained the performance targets related to anaemia treatment.

**Methods:**

The survey was conducted on 3283 patients on haemodialysis (HD) and peritoneal dialysis (PD) at 20 Italian dialysis centres. 540 patients were randomly selected; each centre provided a statistically significant sample proportional to its total number of patients. Maintenance of the following target levels was assessed over time: Haemoglobin (HB) 11-12 gr/dl; Iron: 60-160 mcg/dl; Ferritin: 30-400 mcg/l; Transferrin: 200-360 mg/dl; Transferrin saturation percentage (TSAT %):> 25 <50; Dialysis doses (KT/V): >1.2 <2.0 for non-diabetic HD patients; >1.5 <2.2 for diabetic HD patients; DP: >1.8 <2.5.

Outcome included:

1- Percentage of target maintenance for each parameter.

2- Erythropoietin dose in relation to dialysis techniques, presence of cancer or myeloma, diabetic status, Vitamin B therapy.

3- Erythropoietin dose (International Units/kg/week) (IU/kg/wk) depending on: haemoglobin values, hospitalization of more than 3 days.

**Results:**

Mean age was 65.1; mean haemoglobin concentration over the whole population was 11.3 gr/dl (Standard Deviation (SD): 0.91). The clinical performance targets were maintained over time as follows: HB: 4.3% (Mean 11.43 gr/dl) (SD: 0.42); Ferritin: 71.1% (Mean: 250.23 mcg/L (SD:104.07); Iron: 95.0% (Mean 59.79 mcg/dl)(SD:16.76); Transferrin: 44.8% (Mean 216.83 mg/dl) (SD: 19,50); TSAT %: in 8.4% (Mean: 34.33% (SD: 6.56); HD KT/V: 61.0% (Mean:1.46) (SD: 0.7); PD KT/V:31.4% (Mean: 2.10) (SD: 0.02). The average weekly dose of Erythropoietin (IU/Kg/Wk) was significantly lower for the peritoneal dialysis technique; the higher haemoglobin values, the lower the Erythropoietin dose (IU/Kg/Wk).

**Conclusion:**

A very low percentage of patients maintained haemoglobin target values over time. We need to identify precise criteria to evaluate the stability over time of clinical performance targets proposed by the guidelines.

## Background

Achieving and maintaining stable Haemoglobin levels is a clinical performance target for monitoring the quality and outcome of care in patients undergoing chronic dialysis.

The European Best Practice Guidelines 2004 [1,2] and Italian Guidelines 2003 [3,4] recommend that most patients with Chronic Kidney Diseases (CKD) achieve a target Haemoglobin (Hb) of ≥ 11 gr/dl to reduce the risk of adverse outcomes [5]. The optimal upper Hb level has not been determined [6,7]. Hb target values in the range of 11-12 gr/dl are recommended by the majority of guidelines of other countries, but not all [8-11]. However the current evidence for choosing the minimum Hb target value of 11 gr/dl is very limited. As Kidney Disease Improving Global Outcomes (KDIGO) has yet to publish the new guidelines on anaemia treatment, the European guidelines have maintained the aforementioned target.

Recent studies indicate that Haemoglobin values fluctuate over time [12-15] and that several parameters contribute to Hb cycling, such as: inflammatory or infectious disease processes[16], illness and hospitalisation, fluid balance, iron status and therapy [14,17], Erythropoiesis-stimulating agents (ESAs) dose adjustment [18], management practices and laboratory variation and error [19-21]; moreover, previous studies have yet to pinpoint how to predict the individual response to ESA treatment. Recent studies have also demonstrated that while average Hb levels may fall within desired targets, Hb levels are not consistently maintained within a specified target range over time [13,14,16,22]; furthermore, data indicates that failing to control Hb levels over time may increase the risk of adverse outcomes, including mortality [23-29].

A number of studies have demonstrated the difficulties involved in maintaining Hb levels within a narrow target range. The studies, carried out over variable time periods, involved patients with Chronic Kidney Disease (CKD) in 5th stage conservative therapy [30], or incident or prevalent HD patients [14,16,31]. As simple methods for evaluating and comparing the achievement of performance targets have yet to be standardized, it is difficult to assess their stability in unselected populations of dialysis patients.

For the purpose of evaluating the results and suitability of treatment, a Clinical Audit on anaemia correction on unselected chronic dialysis patients (haemodialysis and peritoneal dialysis) was set up in 2006 in order to assess whether target haemoglobin levels and other clinical performance targets such as KTV, Iron, Ferritin, Transferrin and Transferrin saturation percentage had been obtained and maintained throughout 2005. Some of the results were presented in Ferrara, Italy on 6-7 October 2006 during the Italian Interregional (Emilia Romagna, Tuscany, Liguria) Society of Nephrology Congress.

## Methods

### Study design and data collection

In 2006, twenty Italian dialysis centres in 3 Italian Regions (Emilia Romagna, Tuscany and Liguria) freely participated in a retrospective observational study for a clinical audit on anaemia management in chronic dialysis patients.

The main aim of the study was to evaluate, throughout 2005, how many patients reached and maintained the following performance targets:

• stable Haemoglobin level ≥11 ≤12 gr/dl;

• KT/V:>1.2 <2.0 for non diabetic HD patients;> 1.5 <2.2 for diabetic HD patients; >1.8 <2.5 for PD patients.

• IRON: 60-160 mcg/dl

• FERRITIN: 30-400 mcg/l

• Transferrin: 200-360 mg/dl

• Transferrin saturation percentage: > 25 < 50%

The secondary aim of the study was to evaluate whether:

1. The Erythropoietin doses (IU/kg/wk) were in line with the doses suggested in the European guidelines.

2. There were significant differences between the Erythropoietin doses (IU/kg/wk) administered to HD patients and the doses administered to PD patients.

3. The Erythropoietin doses (IU/Kg/wk) for diabetic patients were significantly higher than those observed in non-diabetic patients.

4. The Erythropoietin doses (IU/Kg/wk) for patients affected by malignancies or myeloma were significantly different from those for non-neoplastic patients.

5. The Erythropoietin doses (IU/Kg/wk) varied significantly in patients on Vitamin B therapy (vitamins B12 and Calcium Folinate).

Target values were established on the basis of the recommendations described in the European (2004) and Italian (2003) guidelines. The clinical performance target for haemoglobin was set between ≥11 ≤12 gr/dl for ≥ 85% of the study population. The observation period covered all patients undergoing chronic dialysis (haemodialysis and peritoneal dialysis) from January 1st, 2005 to December 31st, 2005.

### Selection of patients

#### Criteria for inclusion

Eligible patients had to have undergone peritoneal dialysis or haemodialysis for at least three months during 2005; this included prevalent patients on 31/12/2005, deceased patients or patients who underwent transplant or left dialysis centres after at least three months. The only patients excluded were, therefore, those who had undergone PD or HD for less than 3 months. A random sample, statistically significant at a minimum of 95% within confidence limits and proportional to the total number of patients was taken from each participating centre. The selection criteria were established in agreement with the Office of Medical Statistics of S. Anna Hospital. Patients chosen were identified with a number and, if agreed upon, the first 3 letters of the name or surname so that all data were essentially anonymous; an Excel^® ^spreadsheet for collecting data of random patients was prepared and sent to participating centres.

The following information was collected from medical records: Age, sex, height; start date of dialysis (for incident patients), dialysis type; comorbidities such as diabetes, malignancies, infections, admission to hospital (days); causes of death; monthly body weight; KT/V; weekly Erythropoietin doses; weekly iron therapy; therapy with B-group vitamins (B12 and calcium folinate). Moreover, all haemoglobin, iron, ferritin and transferrin values detected from routine laboratory data throughout 2005 were recorded. Data were put into a database and analysed by the Statistic Medical Service at S. Anna Hospital. Data of incident patients were excluded for the first two months of dialysis treatment. Height and weight were used to calculate body mass index (BMI); the weekly Erythropoietin dose was related to the patient's body weight. For patients receiving Darbopoietin, a conversion factor of 1 μ:200 IU was applied.

### Statistical methods

As it was a retrospective study, it was necessary to resolve some problems.

Target values for each clinical performance parameter were set in accordance with EBPG 2004 and Italian Guidelines 2003. (Table 1) However, no specific criteria (standard) for evaluating the stability of each performance target in relation to the length of the dialysis treatment have yet been identified. As patients were undergoing dialysis for varying periods of time and thus the likelihood that the values would consistently fall within the accepted ranges would decrease proportionally over time, three different patient classes were created in relation to the months of dialysis undertaken during 2005 (Class 1: from 1 to 4 months, Class 2: from 5 to 8 months, Class 3: from 9 to 12 months). Commencing with the assumption that a single value noted outside the stated limits could have a different significance when observed over periods of different lengths, we set arbitrary but reasonable specific intervals of tolerance for each class in relation to the months of dialysis received, in order to evaluate whether the patients maintained the target over time (Table 2). Data from each patient was therefore considered on the basis of: total number of determinations expected for each considered parameter and value of each determination vs. established ranges for each parameter.

**Table 1:** Target values and minimum number of determinations expected for each parameter

Parameter	Minimum number of determination reports within maximum time span	Range considered in statistical processing
**Haemoglobin**	At the beginning & end of the study At least once every 30 days	≥ 11 ≤ 12 gr/dl

**Iron**	At the beginning & end of the study At least once every 60 days	> 15 < 180 mcg/dl

**Transferrin** **TSAT%: > 25 < 50**	At the beginning & end of the study At least once every 120 days	> 170 < 375 mg/dl

**Ferritin**	At the beginning & end of the study At least once every 60 days	> 15 < 700 mcg/l

**KT/V**	At the beginning & end of the study At least once every 30 days for HD patients At least once every 120 days for DP patients	HD* range: >1.2 < 2.0 HD° range: >1.5 < 2.2 DP range: > 1.8 <2,5

*non diabetic patients

° diabetic patients

**Table 2:** Criteria adopted to judge the maintenance of target values over time

Data presence* % Data permanence‡ %	Class 1 from 1 to 4 months of dialysis	Class 2 from 5 to 8 months of dialysis	Class 3 from 9 to 12 months of dialysis
Group 3 "entire permanence"	>= 99%	>= 90%	>= 80%

Group 2 "partial permanence"		>= 85% <90%	>= 75% <80%

Group 1 "limited permanence"		>= 80% <85%	>= 70% <75%

Group -1 "insufficient permanence"	<99%	< 80%	<70%

Percentages of data presence and data permanence within the set limits per parameter in relation to the length of the dialysis treatment

*total number of determinations expected for each considered parameter

‡value of each determination vs. established ranges for each parameter.

The first step was to see whether each patient had the minimum expected number of determinations for each parameter within the set time intervals: for patients who received 1 to 4 months of dialysis (class 1), the number of determinations for each parameter had to be within the expected range for 99% of the time; for those who received 5 to 8 months of dialysis (class 2), the number of determinations had to be within the expected range for more than 90%; and finally, for those patients who received 9 to 12 months of dialysis (class 3), the number of determinations had to be within the range for more than 80% of the time. This was necessary because whilst the majority of Centres noted haemoglobin values at least once a month, 5 centres noted the values of long term dialysis patients only every two or three months (this was however taken into consideration in the 2003 Italian Guidelines). If the number of determinations was sufficient, the percentage of the expected determinations which maintained the target was evaluated. The same percentages were used to judge the maintenance of target values over time, i.e. the data permanence. As a result of this process, based on the percentage of presence and permanence of the data within the limits, at least three ranges of cases were highlighted, and four "permanence" groups were thus created: "entire permanence" (Group 3), "partial permanence" (Group 2), "limited permanence" (Group 1) and "insufficient permanence" (Group -1). In this specific study, only two groups of cases were considered: cases that were always within the set target (Group 3 "entire permanence") and cases that did not reach the planned target (Group -1 "insufficient permanence"); the number of cases belonging to other groups was negligible.

Subsequently, a second stage was set up to answer issues which arose from the first set of results. Parametric/non-parametric procedures such as ANOVA or analogous non-parametric ones: 1 way Analysis of Variance or Notched "Box & Whiskers" test ('BW'), were used to evaluate whether the Erythropoietin dose (IU/Kg/week) was significantly different:

- between the two dialysis techniques (haemodialysis vs. peritoneal dialysis)

- for diabetic patients with respect to non-diabetic patients

- for patients affected by malignancies or myeloma with respect to non-neoplastic patients.

- for patients on Vitamin B therapy (vitamins B12 and Calcium Folinate);

Simple linear regression was used to evaluate possible relationships between Erythropoietin dose (IU/Kg/wk) in relation to haemoglobin values and to hospital admission for more than 3 days.

## Results

In a total study population of 3283 patients, 391 subjects were excluded; of the remaining 2892 eligible patients, 540 were selected for the study. Data from 4 selected patients were excluded as they were deemed insufficient for statistical processing, therefore data from 536 patients were subjected to statistical analysis.

Sample characteristics: 461 patients underwent haemodialysis and 70 patients underwent peritoneal dialysis for the entire period of observation; 5 patients were subjected to both dialysis techniques; 67 patients suffering from diabetes and 44 from malignancies or myeloma, 47 patients died during the observation period. The mean age (male and female) was 65.14; however, the mean age in two centres was more than 71 years. The mean haemoglobin concentration for the whole population was 11.3 gr/dl (SD 0.91).

Mean values of clinical performance targets for patients, who maintained the target for the whole period and all mean values for each parameter in all cases, were obtained as follows (Table 3). All patients (23) who maintained the haemoglobin target over time also held stable iron values (mean value 60.83 mcg/dl) but did not maintain stable Transferrin values or Transferrin saturation percentage. Out of 23 patients, 1 patient with myeloma and 3 patients suffering from malignancies maintained stable haemoglobin levels over time.

**Table 3:** Results

		*Mean values for patients who maintained target for the whole period*				*Mean values for the whole sample*		
**Parameter**	**Total n° of cases**	**Mean value**	**Mean SD**	**Target maintained (cases number)**	**Target maintained %**	**Mean value**	**SD**	**Cases > 0**

**HAEMOGLOBIN** **(g/dl)**	536	11.43	0.42	23	4.3	11.3	0.91	536

**KTV HD ^**	461	1.46	0.7	281	61	1.37	0.06	441

**KT/V PD^°**	70	2.1	0.02	22	31.4	2.02	0.01	51

**IRON** mcg/dl	536	59.79	16.76	511	95	59.75	17.4	531

**FERRITIN** mcg/l	536	250.23	104.07	381	71.1	397.77	148.8	532

**TRANSFERRIN** mg/dl	536	216.83	19.5	240	44.8	187.65	20.42	488

**TRANSFERRIN SATURATION %**	536	34.33	6.56	45	8.4	24.08	7.63	484

**Erythropoietin dose** **IU/Kg/wk**	536	155.21	41.54	400	74.6	143.41	40.87	457

"zero" values are excluded from the calculation of averages.

^ Cases of patients undergoing both dialysis techniques during the observation period were excluded.

° Not all centres provided data.

**Figure 1:**
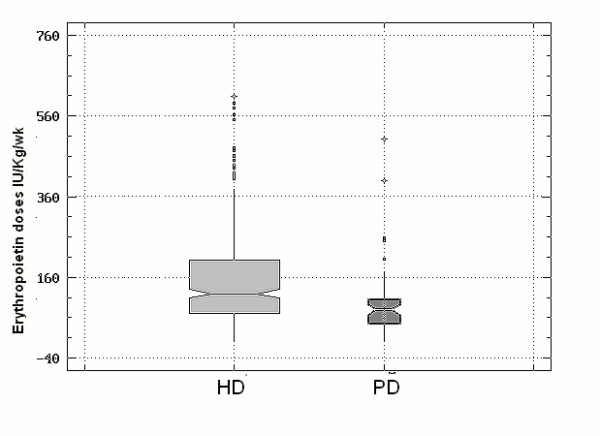
**Erythropoietin doses in relation to dialysis technique**. Cases of patients undergoing both dialysis techniques during the observation period were excluded. CI: Confidence Interval. HD Patients n. 398: Mean Erythropoietin dose IU/Kg/wk: 150.21. (CI: 95%, 139.47:160.94). PD Patients n. 54: Mean Erythropoietin dose IU/Kg/wk: 92.13. (CI: 95%, 63.99:122.27). **P < 0.0003**

Erythropoietin doses were significantly lower for the peritoneal dialysis technique. (P < 0.0003) (Figure 1) but did not vary significantly in relation to diabetes status or use of B12 vitamin and calcium folinate.

**Figure 2:**
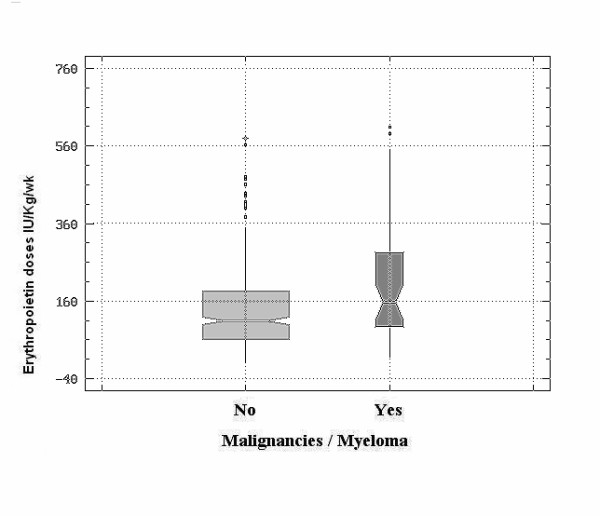
**Erythropoietin doses in patients suffering from malignancies or myeloma vs. non-neoplastic patients**. Patients who developed malignancies or myeloma during the observation period were excluded. CI: Confidence Interval. Patients with Malignancies/myeloma n. 44: Mean Erythropoietin (IU/Kg/wk): 206.75 (CI 95%, 174.70: 238.79). Patients without Malignancies/myeloma n. 413: Mean Erythropoietin (IU/Kg/Wk): 136.66 (CI 95%, 126.20: 147.12). **P < 0.0001**. The averages and medians of Erythropoietin doses seem consistently higher in the group of patients with cancer or myeloma but the difference evaluated by the non-parametric method was not statistically significant.

Erythropoietin doses (IU/Kg/wk) in patients suffering from malignancies or myeloma steadily increased in the group of cancer patients in comparison to the non-neoplastic group but the difference was not statistically significant (Figure 2).

**Figure 3:**
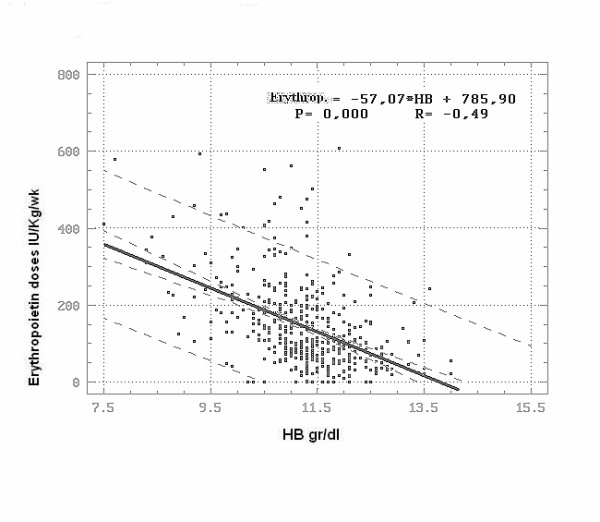
**Erythropoietin doses in dependence on HB values**. Degrees of freedom: 456. Regression coefficient: -57.07. Correlation coefficient (R): - 0.49. Coefficient of determination: 24.48. Regression significance: **P < 0.000**. The regression is greatly significant and the same correlation coefficient is high due to the reduced amount of points distant from the straight line.

A significant decrease in Erythropoietin doses (IU/Kg/wk) was observed with the increase of haemoglobin values (P < 0.000) (Figure 3).

No relationship was found between Erythropoietin doses and hospital admission of more than 3 days, owing to very few cases of hospital admissions of over 4 days.

**Figure 4:**
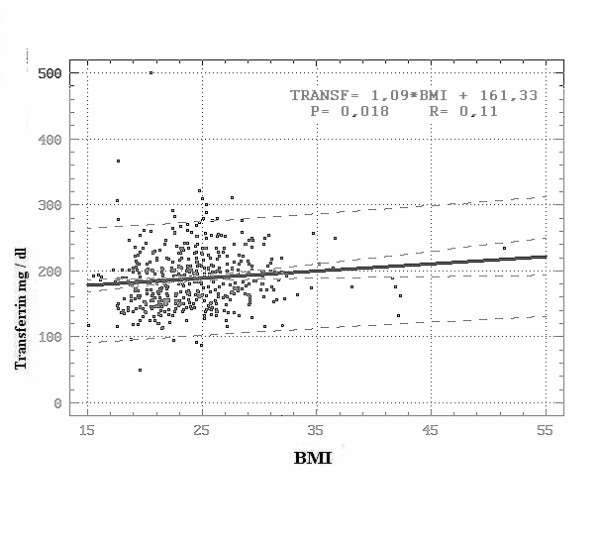
**Transferrin Values in dependence on BMI**. Degrees of freedom: 484. Regression coefficient: 1.09. Correlation coefficient (R): 0.107. Coefficient of determination: 1.15. Regression significance: **P < 0.018**.

As the mean transferrin value was low in a large percentage of patients, a possible relationship between BMI and transferrin was assessed; there appeared to be a rise in transferrin values in relation to increase of BMI (P < 0.018). However, further verifications in terms of higher case numbers and revision of values in selected cases would be needed (Figure 4).

The Transferrin saturation percentage did not show significant variations in relation to BMI.

## Discussion

Reaching and maintaining the standard proposed by the guidelines for long term anaemia treatment in unselected populations of dialysis patients continues to be a very difficult goal to achieve in clinical practice.

Currently, the guidelines recommend reaching and maintaining haemoglobin target values of 11 to 12 gr/dl for the majority of patients in chronic dialysis; the previously (2003) proposed standard requires 85% or more of patients to reach this target.

As the mean value of each parameter calculated during this study did not demonstrate whether the patients had maintained the target over time, a set of arbitrary rules, in relation to the length of dialysis, was created in order to judge the real permanence within the stated limits: the present study, for example, took into account the groups of patients with values constantly within and outside the limits. The adopted method to evaluate the stability of each parameter was arbitrarily set following the European and Italian guidelines, allowing the evaluation of every patient undergoing chronic dialysis during the year in relation to the length of dialysis period (an abstract including early results from this work was previously published [32]).

The method could be criticized because of the percentage of tolerance allowed for defining the maintenance of the target for each parameter, because of the time interval used to define classes of patients or because of the time intervals stated for defining stable Hb values of incident patients.

A positive point, however, is that if it were necessary to modify the fixed limits for each parameter, the percentages could be recalculated without difficulty.

Some factors may have had a negative impact on the results:

1 The decision to not choose specific patients for the population in order to obtain general information.

2 The inclusion of patients who died during the study and patients who underwent less than 6 months of dialysis, as well as the inclusion of patients with malignancies or myeloma.

3 The fact that the population was primarily made up of elderly patients (mean age greater than 65 years), several of whom had limited life expectancy.

This study shows that a very limited percentage of patients maintained the target values for Haemoglobin (4.3%) and Transferrin saturation (TSAT %) (8.4%) over time. The percentage is much lower than the one observed by other authors [16,22,26,29,33] on incident or prevailing patients on haemodialysis.

These results are not, however, strictly comparable, due to the different methods used when carrying out the studies and also due to the different characteristics of populations; results regarding HB targets are similar to those (6.5%) presented by Ebben [14] in HD patients over a six month period.

The mean value for Haemoglobin for the whole population of patients (11.3 gr/dl) does not differ much from the value observed in the population which maintained the target for the whole observation period (11.43 gr/dl).

The mean dose used in subjects who always remained inside target levels was 155.21 IU/kg/wk, versus a mean dose of 143.41 IU used in subjects who always remained outside target levels; the mean HB value leads us to assume that the majority of patients always presented HB levels under 11 gr/dl; transferrin values were stable in 44.8% of cases, while transferrin saturation values were very low, even in presence of iron values that were on target in 95% of cases: this data suggests the need to better evaluate the nutritional status and associated inflammation pathologies.

Moreover, the time required to reach target haemoglobin values for incident patients could have been greater than three months, as this limit was in fact modified by EBPG 2004 and by the Italian Guidelines 2007.

There is now clearly a need to identify precise criteria to evaluate, in clinical practice, the stability of clinical performance targets as proposed by the guidelines.

Using a "mean value" criteria does not define which patients, in the considered population, reach and maintain a standard result over time, thus not enabling one to evaluate if the maintenance of the objective ensures that every patient has a reduced risk of mortality and an improvement in quality of life. The definition of clinical stability criteria is very important when evaluating results gathered during delivery of care to complex patient groups; this also leads to a better use of resources.

## Conclusion

Clinical performance targets were obtained and maintained in different proportions throughout 2005 in the study population.

Data analysis shows that only a small percentage of patients maintained the Haemoglobin target over time and that it is quite clearly a major deviation from the standard set by the guidelines. Transferrin values for many patients for all or part of the observation period are low or very low, suggesting the need of further investigations on the nutritional status or associated inflammatory diseases. The very low percentage of patients maintaining stable Transferrin saturation values despite high percentage of Iron stable values calls for further studies and evaluations.

If the target maintenance is to be regarded as an important goal of care quality, it might be necessary to revise the standard (equal or more than 85%) proposed by the guidelines, especially regarding Haemoglobin, for which the target has to be maintained within narrow ranges.

Simple methods should be identified to enable the verification in clinical practice of the stability over time of clinical performance targets recommended in the guidelines.

## Competing interests

The authors declare that they have no competing interests.

## Authors' contributions

SS, LC and GG were responsible for study conception and design. SC was responsible for acquisition of data. GG analyzed and interpreted the data. SS and GR drafted the manuscript. All authors read and approved the final manuscript.

## Pre-publication history

The pre-publication history for this paper can be accessed here:

http://www.biomedcentral.com/1471-2369/10/33/prepub
